# Implementation of a red blood cell-optical (RBO) channel for detection of latent iron deficiency anaemia by automated measurement of autofluorescence-emitting red blood cells

**DOI:** 10.1038/s41598-020-72382-z

**Published:** 2020-09-24

**Authors:** Takahiro Tougan, Sawako Itagaki, Yuji Toya, Kinya Uchihashi, Toshihiro Horii

**Affiliations:** 1 Research Centre for Infectious Disease Control, Research Institute for Microbial Diseases, Osaka University, 3-1 Yamadaoka, Suita, Osaka 565-0871 Japan; 2 Department of Malaria Vaccine Development, Research Institute for Microbial Diseases, Osaka University, 3-1 Yamadaoka, Suita, Osaka 565-0871 Japan; 3 Sysmex Corporation, 4-4-4 Takatsukadai Nishiku, Kobe, Hyogo 651-2271 Japan

**Keywords:** Parasite host response, Haematological diseases, Infectious diseases

## Abstract

Iron deficiency is the most common and widespread nutritional disorder worldwide. The automated haematology analyser XN-30 (Sysmex, Kobe, Japan) was developed to detect malaria-infected red blood cells (RBCs) in human blood samples using flow cytometry. The optical system of the analyser detects autofluorescence (AF)-emitting RBCs containing iron-deficient haem groups and would aid in the diagnosis of anaemia resulting from iron deficiency. Here, an RBC-optical (RBO) channel was devised and implemented on the analyser. In vitro analyses showed that the analyser detected AF-emitting RBCs treated with 5-aminolevulinic acid. Furthermore, the analyser detected AF-emitting RBCs in mice fed a low iron diet and infected with a rodent malaria parasite; it could also be effectively used in humans. This study demonstrates that the analyser can quantitatively and reproducibly detect AF-emitting RBCs and measure other haematological parameters, suggesting its usefulness for the initial evaluation of latent iron deficiency anaemia in conjunction with the diagnosis of malaria.

## Introduction

Iron deficiency anaemia (IDA) is the most common and widespread nutritional disorder and is estimated to affect approximately two billion people worldwide^1^. In nutritionally poor regions, the diagnosis of IDA is often complicated with infectious diseases such as malaria, parasitic worms, and HIV/AIDS^1^. Because iron deficiency is easily treated with iron supplementation^2,3^, the World Health Organization recommends iron supplementation in conjunction with effective malaria prevention and treatment strategies^4,5^. However, iron administered to women and children without prophylaxis or access to adequate health care is often accompanied by an increase in malaria incidence, thus suggesting that IDA protects against *Plasmodium falciparum* malaria infection^6–11^. This contradiction generates a dilemma for policy makers in dealing with public health management in malaria-endemic areas^12,13^ and makes further clinical studies necessary. Therefore, a rapid and simple assay to detect latent IDA in malaria patients who require additional treatment would be valuable in routine laboratory testing, especially in areas where malaria is endemic and iron deficiency is common.

The automated haematology analyser XN-30 (Sysmex, Kobe, Japan) was developed to quickly detect malaria-causing parasites and to calculate parasitaemia in human blood samples through flow cytometric analysis^14^. In this system, the nucleic acids of malaria-infected RBCs (iRBCs) are stained with a Fluorocell M solution and excited by a semiconductor 405 nm laser beam. Therefore, we expected the optical system of the XN-30 analyser to detect RBCs containing iron-deficient haem (IDH), which is excited with a 402 nm laser and emits at approximately 631 nm^15^.

This study aimed to demonstrate the applicability of the XN-30 analyser for the initial evaluation of IDA in routine laboratory testing. We devised and implemented an RBC-optical (RBO) channel on the analyser and validated detection of autofluorescence (AF)-emitting RBCs in vitro and in vivo. Finally, we assessed the ability of the analyser to detect AF-emitting RBCs in healthy human blood samples.

## Results

### Implementation of the RBO channel on the XN-30 analyser

**Figure 1:**
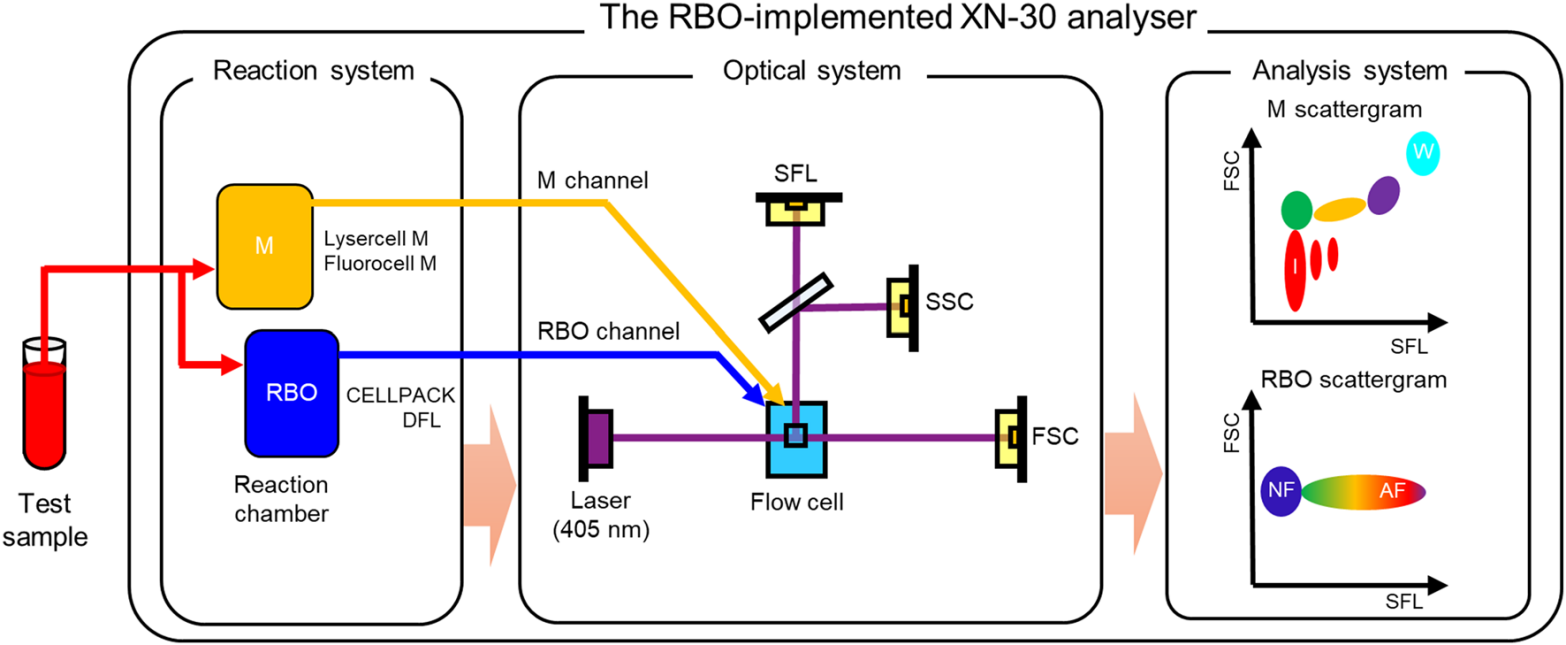
Implementation of the red blood cell (RBC)-optical (RBO) channel on the XN-30 analyser. Test samples are aspirated and separated into two reaction chambers for M and RBO channels in the analyser. Reaction system: in the M chamber (orange), the samples were treated with both Lysercell M and Fluorocell M^14^; in the RBO chamber (blue), the samples were treated with CELLPACK DFL. Optical system: the treated samples were tandemly measured in the same flow cell (light blue) in order of the M and RBO channels. Analysis system: the detected signals were automatically presented on the M and RBO scattergram. “I”, infected RBCs; “W”, white blood cells; “NF”, non-fluorescing RBCs; and “AF”, auto-fluorescing RBCs.

**Figure 2:**
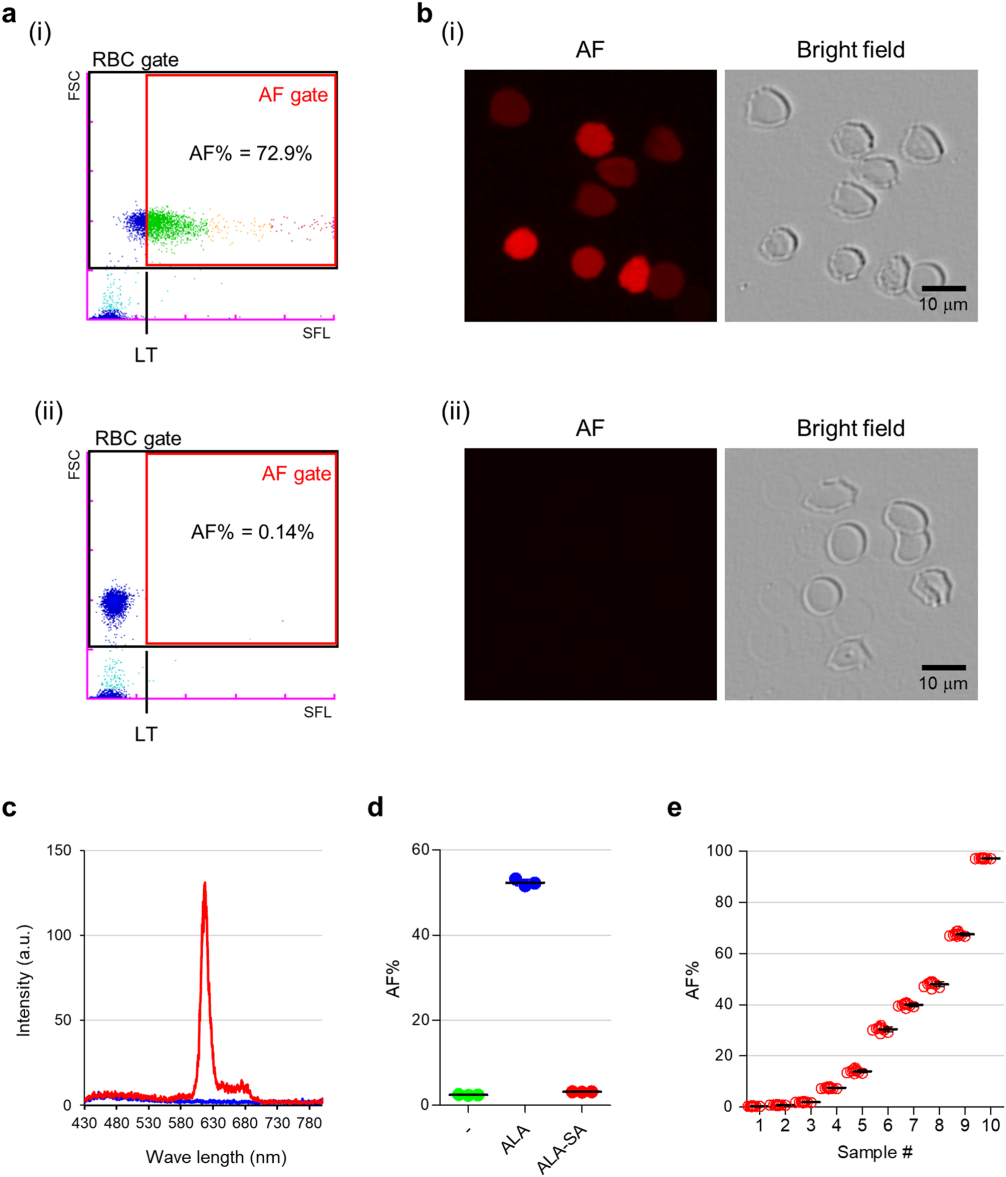
Definition of autofluorescence (AF)-emitting RBCs on the RBO scattergram. (**a**) RBO scattergrams obtained from 5-aminolevulinic acid (ALA)-treated (i) and non-treated (ii) samples. The “RBC gate” and “AF gate” are presented as black and red rectangles, respectively. The forward scattered light (FSC, vertical axis) relates to the cell size and the side fluorescent light (SFL, horizontal axis) indicates the intensity of the AF emitted from RBCs. AF%, AF rate; LT, lower limit. Blue, green, orange, and red dots were arbitrarily coloured according to the intensity of SFL. (**b**) Fluorescence microscopy images obtained from ALA-treated (i) and non-treated (ii) RBC samples. Red represents AF. Scale bar, 10 µm. (**c**) Spectrometry data of the RBC lysates treated with ALA after 72 h. Red and blue lines indicate fluorescence intensities from ALA-treated and non-treated RBC lysates, respectively. Arbitrary units are indicated by “a.u.”. (**d**) AF% of RBCs treated with ALA with/without succinylacetone (SA) after 48 h. “−”, “ALA”, and “ALA-SA” indicate no ALA treatment, treatment with 500 µM ALA, and treatment with 500 µM ALA + 50 µM SA, respectively. Results are expressed as means ± standard error of the mean (SEM) of three individual measurements. (**e**) Reproducibility of measurements with the RBO channel. AF% values are plotted as red open circles. Results are expressed as means ± standard deviation (SD) of ten individual measurements.

To demonstrate that the optical system of the XN-30 analyser can detect AF-emitting RBCs, an RBO chamber/channel was implemented on the XN-30 analyser (Fig. 1). In the analyser, aspirated test samples were separated into two reaction chambers, which feed the analyser’s M and RBO channels. In the M chamber, the samples were treated with both Lysercell M and Fluorocell M^14^; in the RBO chamber, the samples were treated with CELLPACK DFL, which enables the precise determination of RBC size. The treated samples were sequentially measured in the same flow cell in the order of the M and RBO channels. The flow cell was completely washed using CELLPACK DCL after the detection of iRBCs from the M chamber and was used for the detection of AF from RBCs carried from the RBO chamber. Similarly, the flow cell was completely washed using CELLPACK DCL after the detection of AF. In addition, the M and RBO chambers were also completely washed using CELLPACK DCL every measurement. Therefore, the acquired fluorescence was the background level after every measurement (data not shown). The detected signals were automatically displayed on the M and RBO scattergrams as parasitaemias (MI-RBC%) and AF rate (AF%) (Fig. 1, see also Fig. 2a).

### Definition of AF-emitting RBCs on the RBO scattergram

To define AF-emitting RBCs on the RBO scattergram, the signals of both total and AF-emitting RBCs were gated. The forward-scattered light (FSC), shown as the vertical axis, refers to the cell size. Therefore, the upper and lower populations represent RBCs and platelets, respectively (Fig. 2a). In addition, the side fluorescent light (SFL), shown on the horizontal axis, indicates the intensity of the AF emitted from RBCs. The basal gating for counting the number of total RBCs was also determined as the "RBC gate" (Fig. 2a, black rectangle). The vertical axis range for the RBC gate was determined to include most of the RBCs. Specific gating for AF-emitting RBCs (“AF gate”) is indicated on the scattergram (Fig. 2a, red rectangle). The lower limit (LT) of the AF gate along the horizontal axis was determined using the population of RBCs lacking AF in 5-aminolevulinic acid (ALA)-untreated samples as a reference. To determine the LT, 10 ALA-untreated RBC samples were measured, and the average and standard deviation (SD) of the RBC population along the horizontal axis were calculated. The average value plus three-fold SD was defined as the LT of the AF gate. On the vertical axis, the range for the AF gate was identical to that for the RBC gate. The AF% was calculated according to these gates. Fluorescence microscopy confirmed that the observed AF originated from RBCs (Fig. 2b). The spectrometer showed that the emission fluorescence of the RBC lysate excited at 405 nm was most intense at 617 nm (Fig. 2c). This wavelength was nearly identical to that reported in a previous study on PPIX (ca. 631 nm)^15^. In contrast, the generation of AF was disturbed by the addition of succinylacetone (SA), which is an inhibitor of aminolevulinic acid dehydratase in the PPIX synthesis pathway (Fig. 2d). These results suggest that AF was emitted from IDH, and especially from coproporphyrinogen III (CPP) that was generated in the PPIX synthesis pathway (see Discussion). Further, the SD values between measurements were considerably low (SD = 0.080 to 0.93) in each sample (Fig. 2e).

### Characterisation of AF obtained from RBCs

**Figure 3:**
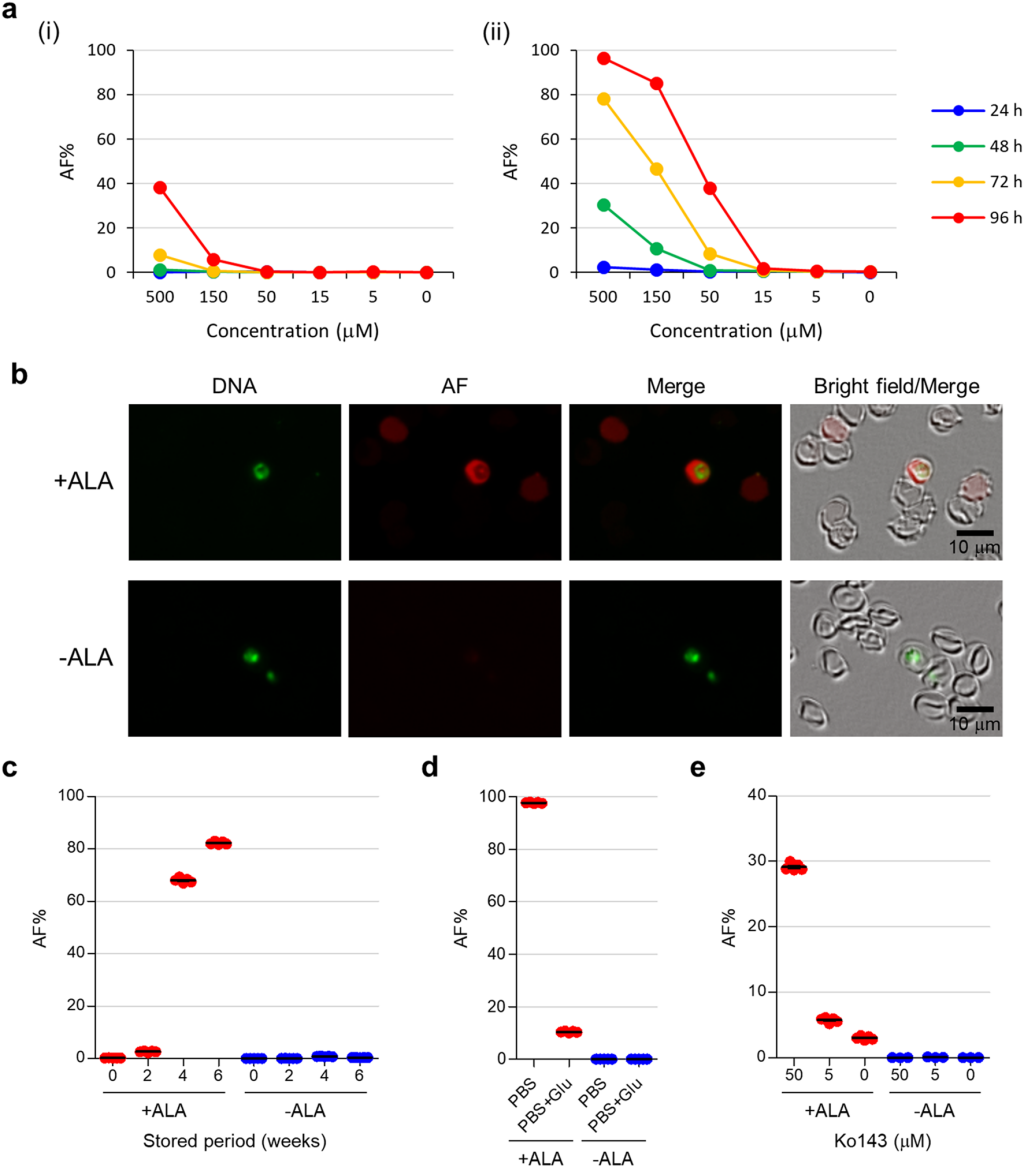
Characterization of the AF generation using the RBO channel. (**a**) AF% of RBCs in non-parasite-culture (i) and parasite-culture medium (ii). The samples were incubated with the indicated concentration of ALA for 96 h. Data represent three independent experiments. (**b**) Fluorescence microscopy of iRBCs after 48 h. Green and red present DNA and AF, respectively. Scale bar, 10 µm. (**c**) AF% of aged RBCs after 24 h. (**d**) AF% of RBCs incubated in PBS with/without glucose after 24 h. “PBS” and “PBS + Glu” indicate PBS and PBS + 4.5 g/L glucose, respectively. (**e**) AF% of RBCs incubated with Ko143 for 72 h. The red and blue dots represent treatment with and without 500 µM ALA, respectively. Results are expressed as means ± SEM of three or five individual measurements.

To characterise the AF obtained from the RBO channel, RBCs were treated with ALA. The AF% of iRBCs was higher than that of RBCs and increased according to ALA concentration and incubation time (Fig. 3a). In addition, the XN-30 analyser also revealed that a high concentration (over 50 µM) of ALA was toxic for parasites (Supplementary Fig. S1). Fluorescence microscopy showed that AF was enhanced not only in iRBCs but also in RBCs (Fig. 3b). This observation indicated that the AF of non-iRBCs was enhanced by co-cultivation with iRBCs, suggesting that changes in culture conditions associated with the growth of the parasite enhanced AF in non-iRBCs.

To determine the factors affecting the enhancement of AF, we examined culture conditions. The analyser showed that stored RBCs increased AF% (Fig. 3c), whereas stored human sera supplemented with culture medium did not affect AF% (Supplementary Fig. S2). Furthermore, the addition of glucose decreased AF% (Fig. 3d), suggesting that a decrease in glucose concentration enhanced AF. In addition, Ko143, an inhibitor of ABCG2 (an exporter of IDH), increased AF% (Fig. 3e), suggesting that ABCG2 exported IDH from the RBCs.

### Evaluation of AF-emitting RBCs and haematological parameters in mice fed a low iron diet (LID)

**Figure 4:**
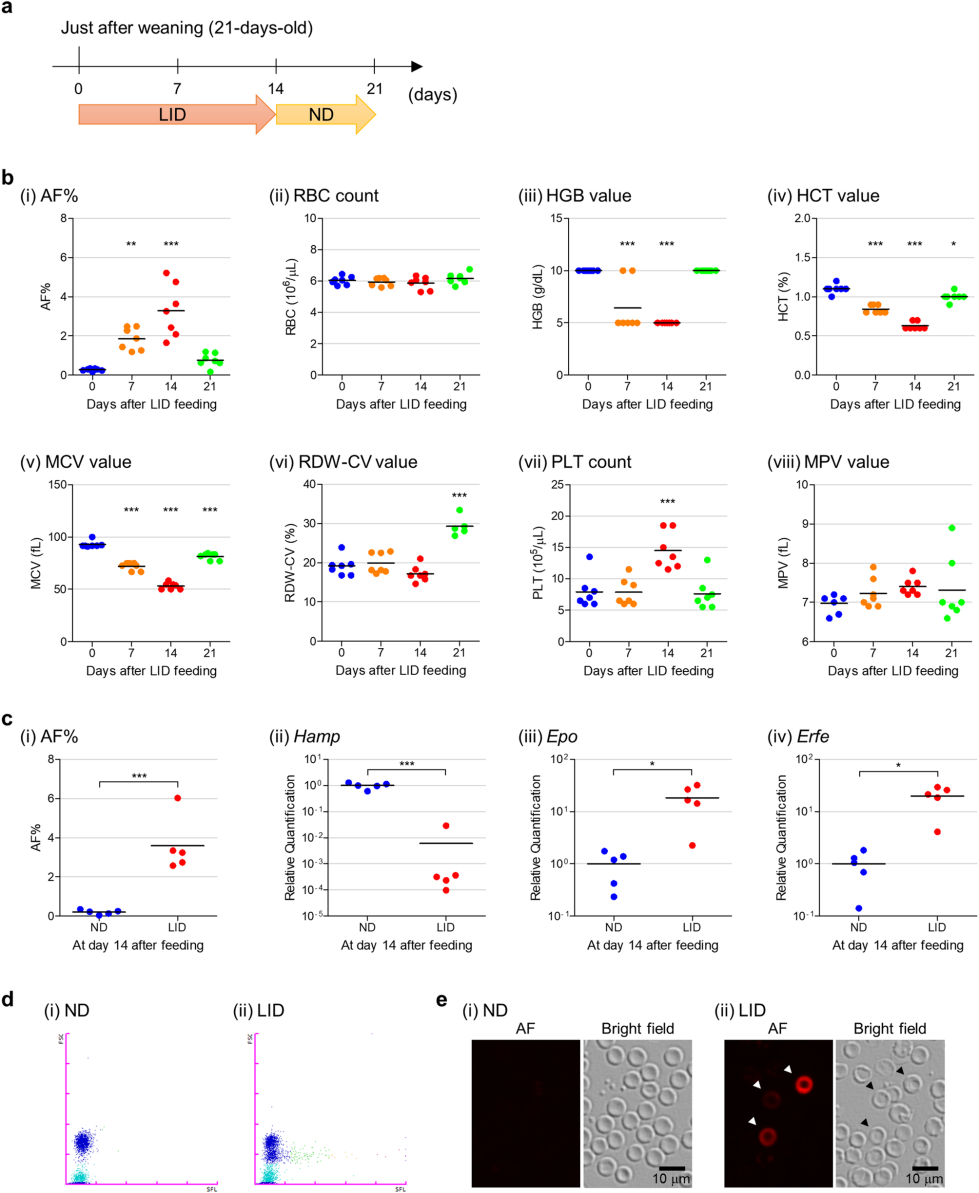
Evaluation of AF-emitting RBCs and haematological parameters in mice fed a low iron diet (LID). (**a**) Schedule of diet-feeding and measurement. Blood samples were collected from mice fed LID and normal diet (ND) and analysed at days 0, 7, 14, and 21. LID and ND were provided during 0 to 14 and 14 to 21 days, respectively. (**b**) Comparison of AF% (i) and haematological parameters: RBC count (ii), haemoglobin (HGB) value (iii), haematocrit (HCT) value (iv), mean corpuscular volume (MCV) value (v), RBC distribution width-coefficient of variation (RDW-CV) value (vi), platelet (PLT) count (vii), and mean platelet volume (MPV) value (viii). Horizontal bar represents the mean. Data were obtained from blood samples diluted at 1:50. The difference between day 0 and days 7, 14, and 21 was analysed statistically. *p < 0.05, **p < 0.01, and ***p < 0.001. (**c**) AF% (i), hepcidin (*Hamp*) mRNA expression level in the liver (ii), erythropoietin (*Epo*) mRNA expression level in the kidney (iii), erythroferrone (*Erfe*) mRNA expression level in the spleen (iv) from mice fed ND and LID at day 14. Horizontal bar represents the mean. The difference between ND and LID groups was analysed statistically. *p < 0.05; and ***p < 0.001. (**d**) RBO scattergrams obtained from mice fed ND (i) and LID (ii) at day 14. The FSC (vertical axis) relates to the cell size and the SFL (horizontal axis) indicates the intensity of the AF emitted by the RBCs. (**e**) Fluorescence microscopy images obtained from mice fed ND (i) and LID (ii) at day 14. Red represents AF. Arrowhead indicates AF-emitting RBC. Scale bar, 10 µm.

To confirm the availability of the RBO channel for the detection of IDA, mice were fed an LID and their blood samples were analysed (Fig. 4a). Mouse AF% increased during feeding (Fig. 4b(i), days 7 and 14); however, AF amounts returned to near basal levels when mice were fed a normal diet (ND) (Fig. 4b(i), day 21). The RBC count remained constant throughout the experiment (Fig. 4b(ii)). Other haematological parameters including haemoglobin (HGB), haematocrit (HCT), and mean corpuscular volume (MCV) values were decreased, whereas platelet (PLT) counts were increased and mean platelet volume (MPV) value was slightly increased at days 7 and 14. These changes were reversed by feeding the ND (Fig. 4b(iii) to (viii) excluding (vi)). In contrast, the RBC distribution width-coefficient of variation (RDW-CV) rate (which shows variation in circulating RBC size) was significantly increased after switching to the ND (Fig. 4b(vi), *p* < 0.001), suggesting the recovery of RBC size (see also Fig. 4b(v)). To confirm that the changes in AF% and haematological parameters were related to haematopoietic responses, we compared mRNA expression levels of hepcidin (*Hamp*) in the liver, erythropoietin (*Epo*) in the kidney, and erythroferrone (*Erfe*) in the spleen. Two weeks after ND- and LID-feeding, the AF% of the LID-fed group was higher than that of the ND-fed group (Fig. 4c(i)). The expression level of *Hamp* mRNA was significantly decreased, whereas the expression levels of *Epo* and *Erfe* mRNA were increased (Fig. 4c(ii) to (iv)). These results suggest that the AF% increase is related to enhanced iron uptake and erythropoiesis^16^. Taken together, these observations suggest that the analyser detects IDA caused by diet, as well as AF-emitting RBCs. In addition, the RBO scattergram showed that AF was mostly emitted from smaller RBCs on day 14 (Fig. 4d), implying that newly generated RBCs are smaller and contain IDH. In contrast, fluorescence microscopy showed that AF was not necessarily emitted from smaller RBCs (Fig. 4e). These results indicate that RBCs treated with CELLPACK DFL, which enables the precise determination of RBC size, are displayed on the scattergram according to their size.

### Evaluation of AF-emitting RBCs and haematological parameters in mice infected with rodent malaria parasite

**Figure 5:**
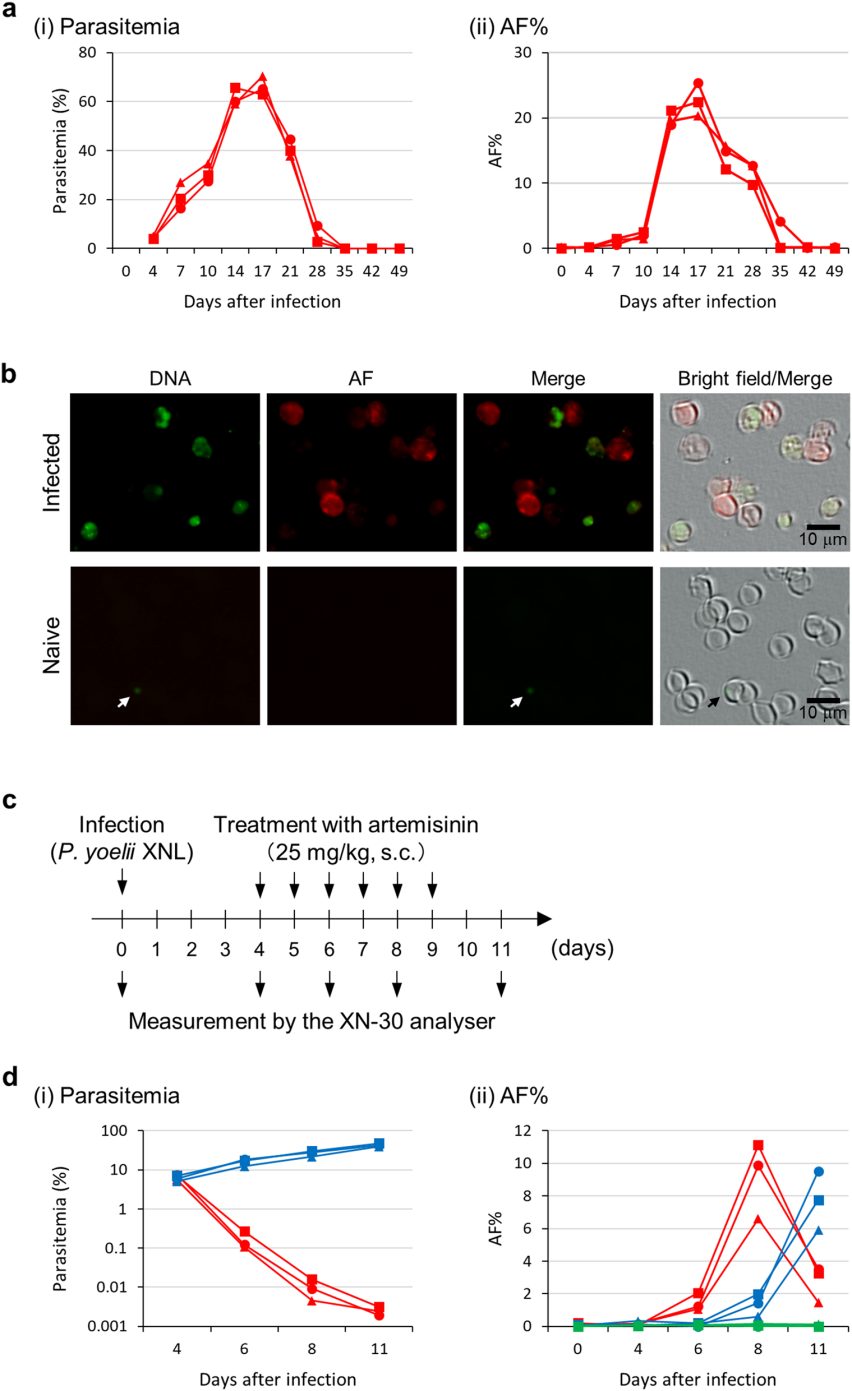
Evaluation of AF-emitting RBCs and haematological parameters in mice infected with the rodent malaria parasite. (**a**) Parasitaemia (i) and AF% (ii) after infection with the *P. yoelii* 17XNL strain. (**b**) Fluorescence microscopy of iRBCs. Green and red indicate DNA and AF, respectively. Arrows indicate Howell-Jolly body. Scale bar, 10 µm. (**c**) Schedule of infection, treatment, and measurement. Artemisinin and solvent were administered subcutaneously at days 4, 5, 6, 7, 8, and 9, as shown by the arrows. (**d**) Parasitaemia (i) and AF% (ii) after infection with the *P. yoelii* 17XNL strain and treatment with artemisinin. Red and blue lines present artemisinin- and solvent-treated mice infected with parasites, respectively. Green lines represent artemisinin-treated naive mice. Data were obtained from blood samples diluted at 1:50. Additional haematological parameters are shown in Supplementary Fig. S3 and S4.

To investigate the relationship between malaria infection and IDA, blood samples collected from parasite-infected mice were analysed. The AF% fluctuated according to the degree of parasitaemia, with changes in AF% appearing after changes in parasitaemia (Fig. 5a), particularly at days 7, 10, and 28. AF% changes resulted from fluctuations not only in RBC counts but also in AF-emitting RBC counts (Fig. 5a and Supplementary Fig. S3). Fluorescence microscopy showed that AF was emitted from non-iRBCs, as well as iRBCs (Fig. 5b). In addition to evaluating changes in AF% that occurred during parasite infection, we also evaluated AF changes after treatment with artemisinin (Fig. 5c). The AF% rapidly increased and then decreased after treatment, in contrast with only an AF% increase observed in the non-treatment group (Fig. 5d and Supplementary Fig. S4). No incremental change in AF% was observed in non-infected mice treated with artemisinin. These results suggest that latent IDA occurs temporarily in parasite-infected mice due to the stimulation of immune/inflammatory responses elicited by artemisinin-induced parasite death.

### Evaluation of AF-emitting RBCs and haematological parameters in healthy humans

**Figure 6:**
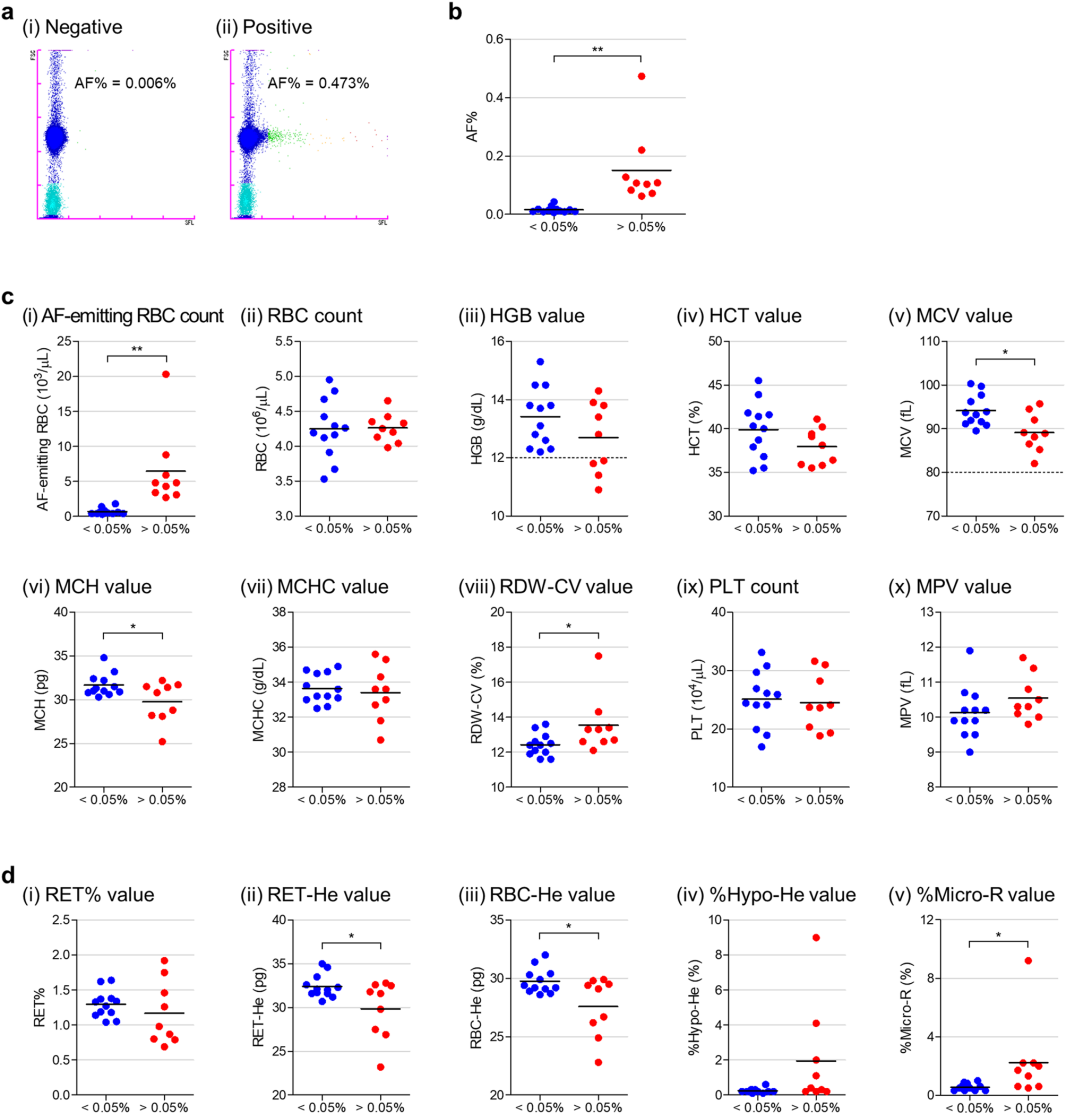
Evaluation of AF-emitting RBCs and haematological parameters in healthy humans. (**a**) Typical RBO scattergrams obtained from human subjects. Less than 0.05 and greater than 0.05 of AF% were defined as negative (i) and positive (ii), respectively. (**b**) Classification of the subjects according to the definition. (**c**) Comparison of haematological parameters obtained from the XN-30 analyser: AF-emitting RBC count (i), RBC count (ii), HGB value (iii), HCT value (iv), MCV value (v), mean corpuscular haemoglobin (MCH) value (vi), mean corpuscular haemoglobin concentration (MCHC) value (vii), RDW-CV value (viii), PLT count (ix), and MPV value (x). The dotted lines at 12 g/dL in HGB panel (iii) and at 80 fL in MCV panel (v) indicate criteria for anaemia and microcytic anaemia, respectively. (**d**) Comparison of haematological parameters obtained from the XN-1000 analyser. Percentage of reticulocytes (RET%) value (i), reticulocyte haemoglobin equivalent (RET-He) value (ii), RBC-He value (iii), percentage of hypochromic RBCs (%Hypo-He) value (iv), and percentage of microcytic RBCs (%Micro-R) value (v). Horizontal bar represents the mean. Data were obtained from undiluted blood samples. The difference between < 0.05% and > 0.05% of AF% groups was analysed statistically. *p < 0.05 and **p < 0.01.

Blood samples from 21 healthy human volunteers were analysed using the XN-30 analyser. We found that the XN-30 analyser could detect AF-emitting RBCs (Fig. 6a). A comparison of AF% allowed for the categorisation of nine subjects as AF-positive (with a threshold of 0.05%). The AF% means were 0.016 and 0.15 in the negative (< 0.05%) and positive (> 0.05%) groups, respectively (Fig. 6b, *p* < 0.01). In comparison with other haematological parameters, the AF-emitting RBC counts of the positive group was significantly higher than that of the negative group (Fig. 6c(i), *p* < 0.01). In addition, the MCV and mean corpuscular haemoglobin (MCH) values of the positive group were significantly lower than those of the negative group (Fig. 6c(v) and (vi), *p* < 0.01), whereas the RDW-CV value of the positive group was significantly higher than that of the negative group (Fig. 6c(viii), *p* < 0.05). Although other parameters did not differ significantly (Fig. 6c(ii) to (iv), (vii), (ix) and (x)), the HGB and HCT values of the positive group were lower than those of the negative group (Fig. 6c(iii) and (iv)). In addition, all four subjects with an HGB value < 12 g/L, which is a criterion of anaemia, were categorised as AF-positive (Fig. 6c(iii)). These data suggest that the subjects in the AF-positive group were anaemic. However, no subjects had an MCV value < 80 fL, which is a criterion of microcytic anaemia with IDA (Fig. 6c(v)). It is not known if IDA patients are included in this tested group, but these observations—using the analysers—indicated a possibility that latent IDA might be present. To corroborate the ability of the XN-30 analyser to suggest the possibility of latent IDA, we evaluated other haematological parameters with the XN-1000 analyser. The XN-1000 analyser revealed that although reticulocyte percentage (RET%) was not significantly different between AF-negative and AF-positive groups (Fig. 6d(i)), the reticulocyte haemoglobin equivalent (RET-He) value of the positive group was significantly lower than that of the negative group (Fig. 6d(ii), *p* < 0.05), suggesting that reticulocytes or immature RBCs contain low haemoglobin content in the positive group. Similarly, the RBC-He value of the positive group was significantly lower than that of the negative group (Fig. 6d(iii), *p* < 0.05), whereas the percentage of hypochromic RBCs (%Hypo-He) and the percentage of microcytic RBCs (%Micro-R) in the positive group were higher than those in the negative group (Fig. 6d(iv) and (v)). These data indicate that reticulocytes and RBCs remarkably contain insufficient haemoglobin and that RBCs are small in the AF-positive group. Taken together, our results suggest that the XN-30 analyser implemented with the RBO channel can help detect latent IDA.

## Discussion

Here, we implemented the RBO chamber/channel on the XN-30 analyser to detect AF-emitting RBCs (Fig. 1). The RBO channel detected AF-emitting RBCs treated with ALA (Figs. 2 and 3). Whereas cancer cells treated with ALA synthesise PPIX in mitochondria, RBCs lacking mitochondria cannot synthesise PPIX^17,18^. Previous studies demonstrated that iRBCs predominantly synthesise CPP, whereas PPIX and coproporphyrin I were synthesised to a lesser extent^17,19^. These findings suggest that AF detected with the RBO channel is predominantly emitted by CPP synthesised from ALA in the cytoplasm of RBCs in the in vitro parasite culture.

RBCs in the parasite culture displayed evident AF (Fig. 3a). Similar enhancement was also observed using stored RBCs (Fig. 3c), in the absence of glucose (Fig. 3d), and upon the addition of an ABCG2 inhibitor Ko143 (Fig. 3e). Although the reason underlying this enhanced accumulation of CPP in RBCs is not fully understood, further studies of this phenomenon would be useful to design applications involving photodynamic diagnosis (PDD) and photodynamic therapy (PDT), as discussed as follows.

Smith and Kain applied ALA to PDT for malaria treatment and found that *P. falciparum* parasite growth was completely inhibited by the application of 200 µM ALA followed by white light exposure or by a higher dose (2 mM) of ALA alone^20^. The current study also demonstrated that the addition of 500 µM ALA partially inhibited parasite growth (Supplementary Fig. S1). However, the direct adoption of ALA-PDT and the use of a high-dose ALA therapy for malaria patients are clinically unrealistic strategies. Recently, Sigala and colleagues showed that the accumulation of CPP in iRBCs could be harnessed for antimalarial chemotherapy using luminol-based chemiluminescence and combinational stimulation with low-dose artemisinin to photoactivate PPIX to produce cytotoxic reactive oxygen^17^. Furthermore, other studies found this pathway to be a promising target for malaria chemotherapy when parasites were treated with ALA supplemented with ferrous sodium citrate in the in vitro* P. falciparum* culture^18^ and in the in vivo rodent malaria model^21^. These studies suggest that RBO technology is useful for follow-up studies involving ALA treatment.

Our feasibility study using the mouse model demonstrated that the analyser was capable of detecting AF-emitting RBCs, as well as haematological parameters and parasitaemia caused by the LID (Fig. 4 and Supplementary Fig. S3) and malaria infection (Fig. 5 and Supplementary Fig. S4). The clinical study demonstrated that the analyser could detect AF-emitting RBCs, as well as haematological parameters (Fig. 6). The recommended initial evaluation of suspected IDA is based on haematological parameters. Especially, RBC-related parameters, including HGB and MCV, are important for the initial evaluation of IDA^22–24^. As the analyser directly detects RBCs containing IDH in addition to these parameters, it is expected that the analyser would not only be useful for the initial evaluation of IDA but would also provide a novel parameter for the diagnosis of IDA. Therefore, blood samples from IDA patients would be required for more direct evidence in further analyses. In addition, two recent studies reported that the XN-30 analyser is available for the study of clinical malaria^25,26^. The XN-30 system can evaluate iRBCs, as well as haematological parameters, in approximately 1 min without the requirement for technical expertise^14^. For the detection of iRBCs, the XN-30 analyser evaluates from approximately 10 thousand to 8 million RBCs/µL every measurement depending on samples. In addition to this, the XN-30 analyser implemented with the RBO channel does not need extra time and specialised handling for the detection of AF-emitting RBCs. These facts suggest that the XN-30 analyser implemented with the RBO channel is useful for the initial evaluation of latent IDA in conjunction with the diagnosis of malaria.

A rapid increase followed by a decrease in the AF% was observed after artemisinin treatment in vivo, suggesting that IDA is also caused by artemisinin treatment (Fig. 5d and Supplementary Fig. S4). It is possible that this rapid change in AF% was caused by a biological response, most likely an inflammatory response elicited by killed parasites. Although previous clinical studies reported delayed haemolysis after artemisinin therapy^27–30^, more in-depth studies regarding the relationship between IDA and antimalarial medicine would also be of clinical importance.

The insertion of iron into PPIX is a pivotal step in the production of haem during erythropoiesis^31,32^. If iron is unavailable, divalent zinc is incorporated into PPIX instead of iron, producing zinc protoporphyrin (ZnPP), which is excited by 405 nm beams and emits at approximately 630 nm, similarly to PPIX^33^. ZnPP persists throughout the life span of RBCs as a biochemical indicator of functional iron deficiency^34,35^. Because our methodology does not distinguish ZnPP from PPIX, the XN-30 analyser implemented with the RBO channel possibly detected RBCs containing ZnPP but not PPIX in the in vivo studies using mouse and human samples.

In the current study, AF% was defined as the ratio of RBCs having an intensity higher than the LT (Fig. 2a). Although this definition distinguished AF-positive from AF-negative RBCs, the difference in AF intensity was not considered (see Supplementary Fig. S5). Utilisation of the AF intensity data, as well as the AF% value, might provide new applications for RBO technology.

In cancer cells, treatment with high concentrations of ALA results in IDH accumulation^36,37^. This accumulation leads to the development of PDD and PDT^38–42^. The principle of AF generation in cancer cells is the same as that in RBCs. Therefore, RBO technology could also have an application in cancer diagnosis and therapy.

In conclusion, the RBO channel-implemented XN-30 analyser quantitatively and reproducibly detected AF-emitting RBCs in vitro. In addition, the analyser detected AF-emitting RBCs in mice fed an LID and infected with rodent malaria parasites; it was also effective for detection in healthy human subjects. Collectively, these results suggest that the XN-30 analyser implemented with the RBO channel would be useful for the initial evaluation of latent IDA in conjunction with the diagnosis of malaria in malaria-endemic and/or non-endemic areas.

## Methods

### Ethics, consent, and permissions

The use of blood samples from healthy Japanese volunteers was approved by the institutional review committees of the Research Institute for Microbial Diseases (RIMD), Osaka University (Approval Number: 22-3) and Sysmex corporation (Approval Number: 2019-07). Informed consent was obtained from all participants. All experimental protocol and methods in this experiment were performed in accordance with the relevant guidelines and regulations from the Declaration of Helsinki. All animal experiments were conducted in accordance with the guidelines of “Animal experiment rules” established by the Research Institute for Microbial Diseases, Osaka University, and were approved by the Animal Care and Use Committee of the Research Institute for Microbial Diseases, Osaka University (Approval Numbers: Biken-AP-H26-06-0 and Biken-AP-R01-03-0).

### Compounds

ALA was purchased from Cosmo Bio (Tokyo, Japan) and prepared as a 100 mM stock solution in saline (0.9 g/L NaCl). SA was obtained from Cayman Chemicals (Ann Arbor, MI, USA) and prepared as a 10 mM stock solution in saline. Ko143 was purchased from Sigma-Aldrich (St. Louis, MO, USA) and prepared as a 10 mM stock solution in dimethyl sulfoxide (DMSO).

### The automated haematology analyser, XN-30

An XN-30 analyser (Sysmex) equipped with a prototype algorithm for cultured *P. falciparum* parasites (software version: 01-03, (build 16)) was used for the detection of malaria parasites. In brief, the XN-30 analyser aspirated and diluted blood samples in a CELLPACK DCL solution at a specific dilution ratio (1:50). Subsequently, nucleic acids were stained with a staining solution (Fluorocell M) along with a lysis solution (Lysercell M), and iRBCs and WBCs were excited by a 405 nm laser beam. Approximately 100 µL of the culture suspension diluted with 100 µL of phosphate-buffered saline was added to an EDTA-K_2_ microtube (BD Microtainer MAP microtube for automated process; Becton Dickinson and Co., Franklin Lakes, NJ, USA) and loaded in the XN-30 analyser with an auto-sampler, as described in the instrument manual (Sysmex). The XN-30 analyser generated an M scattergram, which showed the developmental stages of the parasites according to DNA content and iRBC size^14^. The parasitaemias (total, MI-RBC%; ring-form, RNG-RBC%; trophozoite, TRPZ-RBC%; and schizont, SCHZ-RBC%) were reported automatically.

### Implementation of the RBO channel on the XN-30 analyser

The RBO channel was implemented on the XN-30 analyser. The test samples were injected into the RBO chamber and then treated with CELLPACK DFL. The treated samples were measured in the flow cell in order of the M and RBO channels. For the RBO channel, approximately 60 thousand RBCs were evaluated in this study of the human peripheral blood sample. The analyser simultaneously provided an RBO scattergram, in which the dots were presented according to AF intensity and cell size (see Fig. 1). Horizontal and vertical axes represented the intensities of RBO-side fluorescent light (SFL, indicating the intensity of the AF) and forward scattered light (FSC, indicating RBC size), respectively. “RBC gate” and “AF gate” were configured according to the respective intensities (see Fig. 2a). The AF% was calculated according to Eq. (1):

1 $${\text{AF}}\% = \left( {\# {\text{dots}}\;{\text{in}}\;{\text{AF}}\;{\text{gate}}} \right)/\left( {\# {\text{dots}}\;{\text{in}}\;{\text{RBC}}\;{\text{gate}}} \right) \times {1}00$$

### Treatment of RBCs with ALA, SA, and/or Ko143 in vitro

For in vitro analyses, RBCs and ring-form-synchronized iRBCs were prepared in parasite culture medium or in PBS with the indicated concentration of ALA, SA, and/or Ko143 and incubated under defined culture conditions (see the section: Parasite strain and culture in vitro). In in vivo analyses, the addition of ALA is not required because IDH is generated in response to diet and malaria infection.

### Parasite strain and culture in vitro

*P. falciparum* laboratory strain 3D7 was obtained from Prof. Masatsugu Kimura (Osaka City University, Osaka, Japan). For the assessment of antimalarial activity of the compounds in vitro, the parasites were cultured in RPMI 1,640 medium (containing 2.0 g/L glucose) supplemented with 0.5 g/L L-glutamine, 5.96 g/L HEPES, 2 g/L sodium bicarbonate (NaHCO_3_), 50 mg/L hypoxanthine, 10 mg/L gentamicin, 10% heat-inactivated human serum, and RBCs at a 3% haematocrit level in an atmosphere of 5% CO_2_, 5% O_2_, and 90% N_2_ at 37 °C, as previously described^43^. RBCs infected with ring-form parasites were collected using the sorbitol synchronisation technique^44^. Briefly, the RBCs were collected by centrifugation at 840 g for 5 min at room temperature, suspended in a fivefold volume of 5% D-sorbitol (Nacalai Tesque) for 10 min at room temperature, and then washed twice with RPMI 1,640 medium to remove the D-sorbitol.

### Diet-induced IDA

Experimental IDA was induced by a LID^16^. Briefly, female ICR mice (21 days-old) were purchased from Japan SLC (Shizuoka, Japan). For induction of IDA, 7 mice were fed a LID containing 3.6 ppm Fe. As a control, CLEA Rodent Diet CE-2, which contains 310.2 ppm Fe, was used as the ND. Both diets were obtained from CLEA Japan (Tokyo, Japan). The animals were fed a LID for 14 days, after which a ND was given for 7 days.

### Quantitation of messenger RNA (mRNA) levels

Total RNA was harvested from mouse liver, kidney, and spleen with the RNeasy Mini Kit (Qiagen, Hilden, Germany). Complementary DNA was synthesized using the High Capacity cDNA Reverse Transcription Kit (Applied Biosystems, Foster City, CA, USA). Quantitative polymerase chain reaction (qPCR) was performed with Luna Universal qPCR Master Mix (NEB, Hitchin, UK) and run in triplicate on the Illumina qPCR Eco system (Illumina, San Diego, CA, USA). Expression levels of *Hamp*, *Epo*, and *Erfe* mRNA were normalized to the reference 60S ribosomal protein L4 (*Rpl4*), hypoxanthine phosphoribosyl transferase (*Hprt*), and *Rpl4* mRNA, respectively^45^. Data were expressed as relative quantification, i.e. comparison between ND and LID mice. Primer sequences are indicated in Table 1.

**Table 1:** Primer sequences for quantitative polymerase chain reaction (qPCR).

Gene name	Forward	Reverse
*Hamp*	AAGCAGGGCAGACATTGCGAT	CAGGATGTGGCTCTAGGCTATGT
*Epo*	GCCTCACTTCACTGCTTCGG	GGAGGCGACATCAATTCCTTC
*Erfe*	ATGGGGCTGGAGAACAGC	TGGCATTGTCCAAGAAGACA
*Rpl4*	TGAAAAGCCCAGAAATCCAA	AGTCTTGGCGTAAGGGTTCA
*Hprt*	CTGGTTAAGCAGTACAGCCCCAA	CAGGAGGTCCTTTTCACCAGC

### Parasite, infection, and drug treatment in a mouse model

Female C57BL/6 mice (6 weeks-old) purchased from Japan SLC were injected intraperitoneally with 3 × 10^5^ iRBCs with the non-lethal strain *P. yoelii* 17XNL. Three mice were analysed for analysing the parasite infection (Fig. 5a). For the analysis of parasite infection and treatment (Fig. 5d), 6 parasite-infected mice (3 mice per group) and 3 non-infected mice were analysed; 3 infected mice and 3 non-infected mice were treated with artemisinin. A stock solution of 50 mg/mL of artemisinin (TCI, Tokyo, Japan) was prepared (65% DMSO and 35% Tween-80) and then further diluted to 5 mg/mL (1:10 dilution) with saline. The mice were subcutaneously administrated with artemisinin (25 mg/kg body weight) 4 days after infection with the *P. yoelii* 17XNL parasite^46^. Blood samples were collected before and after drug treatment and analysed with the XN-30 analyser. Briefly, row data (saved as FCS file) were exported from the XN-30 analyser and analysed by the Flowing Software 2.5.1 (Turku Centre for Biotechnology, University of Turku, Turku, Finland) using three-dimensional analysis^40^. RBC count, HGB value, PLT count, and WBC count were calculated according to the indicated dilution ratio (1:50), while HCT, MCV, and MPV values were used directly^40^.

### Fluorescence microscopy

Nucleic acids were stained with 20 nM coriphosphine O (CPO) (TCI, Tokyo, Japan) for 5 min at room temperature and then washed with saline once by centrifugation at 840 g for 5 min at room temperature. Images were captured using a BZ-X710 fluorescence microscope (Keyence, Osaka, Japan). A specific filter (5ALA-405UF1-BLA; excitation = 405/20 nm, emission = 640/30 nm and dichroic mirror = 425 nm) and a BZ-X GFP filter (OP-87763; excitation = 470/40 nm, emission = 525/50 nm and dichroic mirror = 495 nm) were used for detection of AF and CPO, respectively.

### Measurement of the fluorescence spectrum

RBCs were treated with 500 µM ALA (dissolved in DMSO) or with DMSO in RPMI 1,640 medium under the parasite culture condition for three days. Treated RBCs were collected by centrifugation at 200 g for 5 min at 4 °C. The collected RBCs were disrupted in water and centrifuged at 200 g for 5 min at 4 °C. The supernatants were measured with an F-4500 fluorescence spectrophotometer (Hitachi, Tokyo, Japan). The samples were excited at 405 nm (bandpass width, 5.0 nm) and their emission spectra were recorded between 200 and 800 nm.

### Measurement of human blood samples

Twenty-one healthy Japanese female volunteers participated in the RBO analysis. Peripheral blood samples were collected by EDTA-K_2_ tube (Terumo, Tokyo, Japan) and were analysed in the XN-30 and XN-1000 analysers within 4 h after blood collection without any dilution. The XN-1000 analyser provided data of RET%, RET-He, RBC-He, %Hypo-He, and %Micro-R values for evaluation of anaemia^47,48^.

### Statistical analyses

The statistical significance of differences between groups of the LID-fed mice was evaluated through a one-way analysis of variance followed by Tukey’s multiple comparison tests using GraphPad Prism version 5.0 (GraphPad Software, San Diego, CA, USA). Statistical significance of differences between two groups in the other experiments was calculated by an unpaired two-tailed Student's *t* test using GraphPad Prism version 5.0.

## Supplementary information

Supplementary Information 1

## Data Availability

The datasets used and/or analysed during the current study are available from the corresponding author upon reasonable request.
