# Supplementary material for: Implementation of a red blood cell-optical (RBO) channel for detection of latent iron deficiency anaemia by automated measurement of autofluorescence-emitting red blood cells
Source: Sci Rep. 2020 Sep 24;10:15605. doi: 10.1038/s41598-020-72382-z (PMC7518259; doi:10.1038/s41598-020-72382-z)
Supplement: Supplementary file 1 — Supplementary Information 1 [file 41598_2020_72382_MOESM1_ESM.docx]

Supplementary Information

**Implementation of a red blood cell-optical (RBO) channel for detection of latent iron deficiency anaemia by automated measurement of autofluorescence-emitting red blood cells**

Takahiro Tougan^1^*, Sawako Itagaki^2^, Yuji Toya^3^, Kinya Uchihashi^3^, Toshihiro Horii^2^

^1^ Research Centre for Infectious Disease Control, Research Institute for Microbial Diseases, Osaka University, 3-1 Yamadaoka, Suita, Osaka 565-0871, Japan

^2^ Department of Malaria Vaccine Development, Research Institute for Microbial Diseases, Osaka University, 3-1 Yamadaoka, Suita, Osaka 565-0871, Japan

^3^ Sysmex Corporation, 4-4-4 Takatsukadai Nishiku, Kobe, Hyogo, 651-2271, Japan

*Corresponding author: [ttougan@biken.osaka-u.ac.jp](mailto:ttougan@biken.osaka-u.ac.jp)

**Supplementary Fig. 1.** Efficacy of ALA on parasite development.

Parasitaemia (MI-RBC%) after ALA-treatment. Parasites were incubated for 48 h. Results are expressed as means ± SEM of five individual measurements.

**
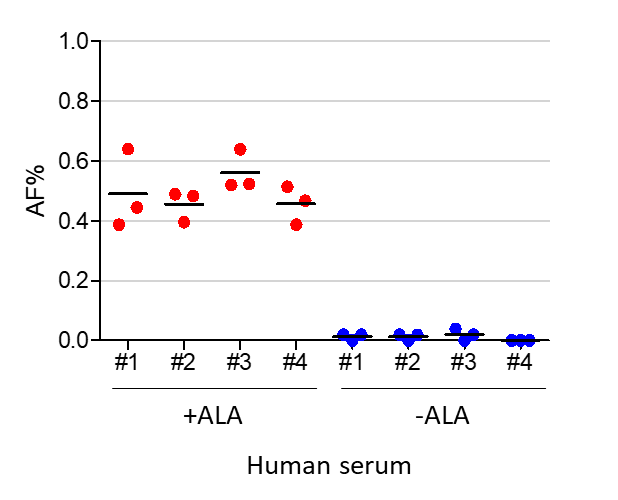
**

**Supplementary Fig. 2.** Effect of human serum on AF emission.

AF% of RBCs incubated in culture medium added to four types of human serum for 48 h. Results are expressed as means ± SEM of five individual measurements.

**
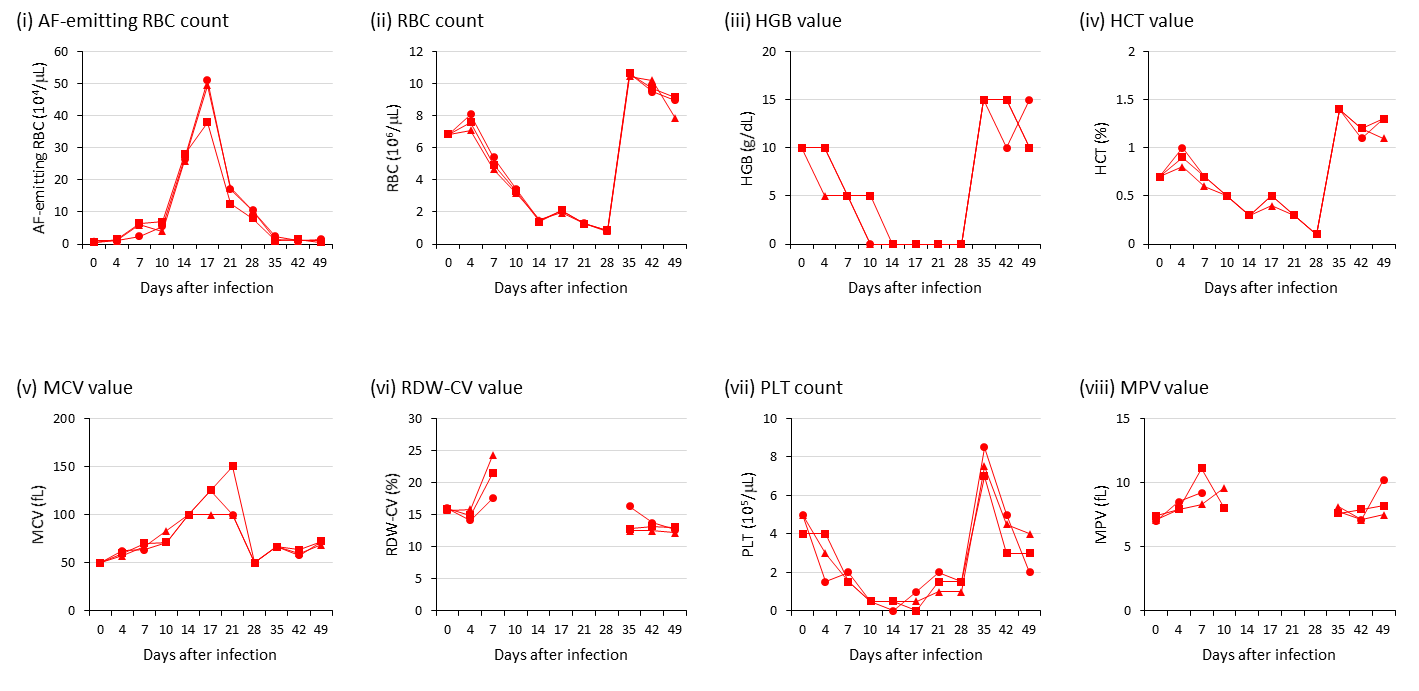
**

**Supplementary Fig. 3.** Haematological parameters after parasite infection.

Haematological parameters after infection with *P. yoelii* 17XNL strain. AF-emitting RBC count (i), RBC count (ii), HGB value (iii), HCT value (iv), MCV value (v), RDW-CV value (vi), PLT count (vii), and MPV value (viii). No RDW-CV and MPV values (vii) are out of the XN-30 analyser range.

**
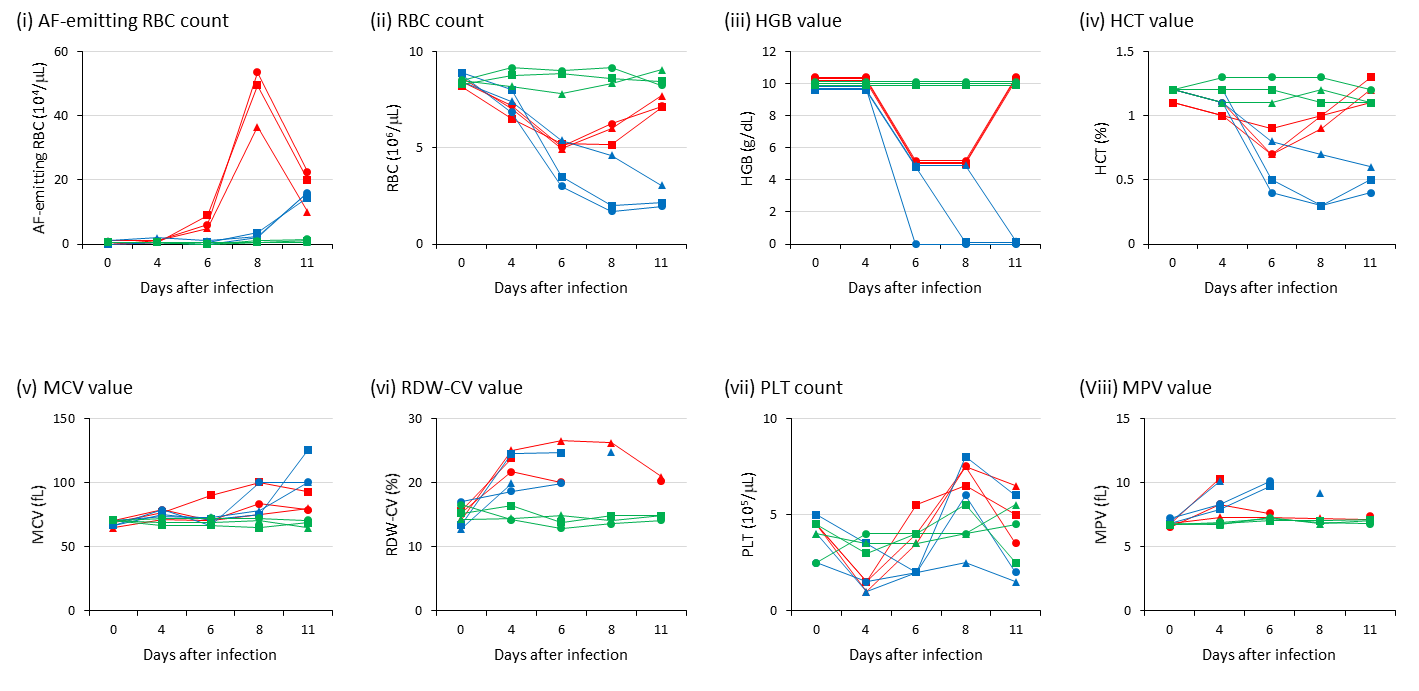
**

**Supplementary Fig. 4.** Haematological parameters after infection and treatment with artemisinin.

Haematological parameters after infection with the *P. yoelii* 17XNL strain and treatment with artemisinin. AF-emitting RBC count (i), RBC count (ii), HGB value (iii), HCT value (iv), MCV value (v), RDW-CV value (vi), PLT count (vii), and MPV value (viii). Red and blue lines represent anti-malarial drugs and solvent-treated mice infected with parasites, respectively. Green lines represent artemisinin-treated naive mice. Anti-malarial drugs and solvent were administered subcutaneously at days 4, 6, and 8. Data were obtained from blood samples diluted at 1:50.

**
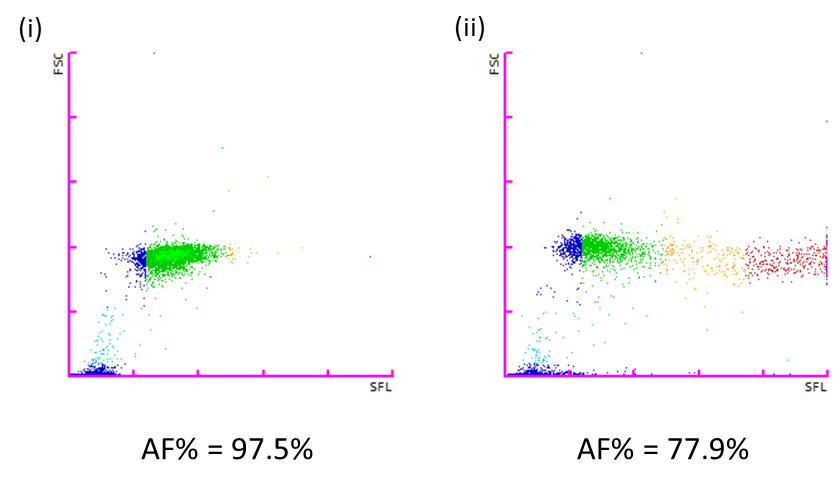
**

**Supplementary Fig. 5.** RBO scattergram of RBCs treated with ALA.

RBO scattergrams with different AF% and AF intensity. Values for AF% were 97.5% (i) and 77.9% (ii). The FSC (vertical axis) relates to the cell size and the SFL (horizontal axis) indicates the intensity of the AF emitted from RBCs. Blue, green, orange, and red dots were arbitrarily coloured according to the intensity shown by the SFL. Blue dots indicate non-fluorescing RBCs, whereas green, orange, and red dots indicate fluorescing RBCs.
